# Efficacy and Safety of Ejiao (Asini Corii Colla) in Women With Blood Deficient Symptoms: A Randomized, Double-Blind, and Placebo-Controlled Clinical Trial

**DOI:** 10.3389/fphar.2021.718154

**Published:** 2021-10-11

**Authors:** Li Zhang, Zhongju Xu, Tao Jiang, Jialu Zhang, Pinxian Huang, Jiaqi Tan, Gan Chen, Man Yuan, Zhuo Li, Haibin Liu, Dengfeng Gao, Lianbo Xiao, Hui Feng, Jiatuo Xu, Hongxi Xu

**Affiliations:** ^1^ Institute of Arthritis Research, Guanghua Hospital Affiliated to Shanghai University of Traditional Chinese Medicine, Shanghai, China; ^2^ School of Pharmacy, Shanghai University of Traditional Chinese Medicine, Shanghai, China; ^3^ School of Basic Medicine, Shanghai University of Traditional Chinese Medicine, Shanghai, China; ^4^ National Engineering Research Center for Gelatin-based Traditional Chinese Medicine, Dong-E-E-Jiao Co. Ltd., Done-E Country, China

**Keywords:** asini corii colla (Ejiao), blood deficient symptoms, randomized controlled trial, anemia, TCM syndromes

## Abstract

*Equus asinus* L [Equidae; Asini Corii Colla] (donkey-hide gelatin, Ejiao), a well-known traditional Chinese medicine, has been widely used to nourish the blood, especially for women. The aim of this study was to assess the efficacy and safety of Ejiao in blood-deficient patients. A total of 210 participants were recruited and randomly allocated into the placebo control group and Ejiao-treated group (6 g/day). The primary outcomes on the efficacy of Ejiao included traditional Chinese medicine symptom scores, blood indicators, and SF-36. The secondary outcomes were changes in fireness and safety evaluation. Results showed that Ejiao treatment for 8 weeks had significantly improved dizziness symptoms. Among the tested 24 blood biochemical parameters, the hematocrit and red blood cell numbers decreased in the placebo control group, but decreased significantly less in the Ejiao treatment group. The white blood cell and neutrophil counts increased in the Ejiao group but were within the normal range. In addition, the quality of life improved as the scores in SF-36 domains were significantly higher in the Ejiao group. At the same time, there was no significant change in the fire–heat symptoms score or other safety parameters. Considering all these, our study showed that Ejiao has a promising effect in women suffering from blood deficiency without obvious adverse effects.

## Introduction

Blood deficiency syndrome (BDS) is one of the common clinical syndromes of traditional Chinese medicine (TCM), which refers to the pathological state of insufficient blood. The main clinical symptoms are dizziness, pale complexion, and reduced menorrhagia in women. BDS in TCM is not exactly the same as in anemia in modern medicine, although both of them refer to the reduction of blood cells or hemoglobin. BDS is often associated with impaired hematopoietic function, peripheral blood pancytopenia, hypovisceral dysfunction, malnutrition, and even myelosuppression (Zhang et al., 2014; Ji et al., 2018; Zhang et al., 2020). Therefore, BDS is related to but different from anemia in modern medicine.

It has been reported that many traditional Chinese medicine showed curative effects on BDS (Wu J. et al., 2007; Zhang et al., 2014; Ji et al., 2018). Ejiao is an ancient traditional Chinese medicine, which is prepared by stewing and concentrating from *Equus asinus* L. donkey hide. Ejiao has been used for more than 2000 years in China, and the main components have been collagen and amino acids (Du et al., 2019). According to the ancient books, the major functions of Ejiao were tonifying blood and nourishing Yin, which have been considered as the first choice for the treatment of BDS. Nowadays, Ejiao has been widely used in the clinic for its biological activities of anti-fatigue, immunity improvement, tumor suppression, and specifically anti-anemia effect (Wu H. et al., 2007; Chen et al., 2012; Wang et al., 2012; Liu S. et al., 2014; Zhang et al., 2018). The hematopoietic effect of Ejiao has also been approved by modern biological studies, and it was reported that the fractions from Ejiao promoted hematopoiesis on mice with 5-fluorouracil–induced anemia (Wu H. et al., 2007). RNA-sequencing studies have indicated that the molecular mechanisms of Ejiao might be related to the extracellular matrix–receptor interaction, Wnt signaling, and PI3K-Akt signaling pathway (Zhang Y. et al., 2019). However, most clinical studies are focused on the formula that comprises Ejiao, such as the *Fufang E’jiao Jiang* that has been reported to improve the hematopoietic functions and increase the Hb level of postpartum anemic women rapidly and significantly (Li et al., 2018). However, the clinical efficacy of Ejiao alone has not been studied systemically.

Ejiao has been used for thousands of years, and the demand for it is increasing every year. However, there have always been concerns about “fireness” induced by improper use of Ejiao. Fireness is a traditional syndrome of Chinese medicine without specific physiological indicators (Chong and Oberholzer, 1988; Zhang M. et al., 2019). According to the TCM theory, fireness refers to the hot symptoms in the human body which are caused by an imbalance of Yin and Yang. The specific symptoms include red and swollen eyes, erosion at the corners of mouth, yellow urine, toothache, sore throat, etc. (Xiao and DeFranco, 1997; Wu J. et al., 2007). In the clinic, fireness is mainly diagnosed according to the feelings of patients. Although many people thought Ejiao might cause fireness, there has been no systematic study on it. In our study here, the double blind and randomized clinical trial was carried out to evaluate the effect of Ejiao on patients with BDS. The aim of the present study was to evaluate the safety, particularly the side effects of “fireness,” and the anti-fatigue effect of Ejiao on patients with deficiency syndrome.

## Materials and Methods

### Participants

This study was designed as a randomized, double-blind, placebo-controlled study according to the CONSORT 2017 statement (Cheng et al., 2017). The participants were recruited from March 2019 to December 2020 at Guanghua Hospital Affiliated to Shanghai University of Traditional Chinese Medicine (Shanghai, China).

In this study, the inclusion criteria were as follows: 1) the syndrome differentiation of TCM is BDS; 2) males or females aged 18–60 years; 3) no anti-anemia foods or drugs used within 3 months; and 4) without any severe disease of the heart, liver, kidneys, or blood system.

The exclusion criteria included the following: 1) patients with severe anemia (Hb < 90 g/L); 2) acute hemorrhage and hemolysis; 3) acute infection, acute stage of a chronic disease; 4) lactating or pregnant women; 5) not suitable for Ejiao treatment according to their physician; 6) unable to follow dietary or drug restrictions; and 7) participation in another clinical trial within the past 3 months.

The sample size was calculated using PASS 15. A total sample size of 177 provided a test power of 0.8 to detect the difference by using a two-sided t-test when the significance level was 0.05. We utilized a parallel-controlled design with an equal number of participants in each group. However, considering a 11 and 15% withdrawal rate in the control and Ejiao groups, respectively, 210 participants were recruited, and 181 participants completed the study (Chow et al., 2008).

All participants submitted an informed consent before the start of the study. This study was approved by the Institutional Review Board of Guanghua Hospital Affiliated to Shanghai University of Traditional Chinese Medicine (No. AF13v2-2). The clinical trial has been registered at the Chinese Clinical Trial Registry with the registration number: ChiCTR-TRC-1900021651.

### Study Design

Those patients who meet the inclusion criteria were randomly assigned to receive either Ejiao or placebo at a 2:1 ratio. Randomization was performed by a third party responsible for sequentially assigning random numbers and distributing trial samples to the patients. The patients received capsules either with Ejiao or placebo, 6 g each day. Both the Ejiao capsules were manufactured by the company Dong-E-E-Jiao Co. Ltd. These capsules are identical in size, weight, color, and taste. The researchers and patients did not know which group the patients are allocated to from the appearance of the medication given.

### Outcome Measurements

The primary outcome included the change in the total score of the Blood Deficiency Symptoms Grading and Quantifying scale (BDS scale). Based on the Guideline of Clinical Research of TCM New Drugs (Zheng, 2002), the BDS scale consists of the following seven items categorized as major and secondary symptoms: 1) pale complexion; 2) dizziness; 3) palpitations; 4) pale tongue; 5) thready pulse; 6) limb numbness; and 7) insomnia. The items from one to five are the main symptoms; the other items are the secondary symptoms. Each symptom is directly transformed into a 0–6 scale based on the severity of the disease. The instrument yields a summed total score ranging between 0 and 30 (0 = no symptoms).

### Secondary Outcomes

The secondary outcomes included the changes from the baseline in blood cells; the quality of life that is assessed by a change in the Short Form 36 scale score (Ware and Sherbourne, 1992; Steinhaus et al., 2019; Chen et al., 2020).

### Safety Assessments

Safety assessments included the fire–heat symptom scale, routine examination, and adverse events (AEs).

The fire–heat symptom scale was used to determine fireness in this study as had been described in our previous study (Zhang M. et al., 2019), which includes excess fire–heat (29 items) and deficiency fire heat (14 items) (Lin et al., 2012; Liu M. et al., 2014). Routine examination (blood and urine routine examination, liver and renal functions, and electrocardiogram) were carried out at the baseline and at the end of the study. AEs were monitored throughout the study to assess the safety of Ejiao.

### Statistical Methods

Analyses of efficacy were performed on the intent-to-treat population (full analysis set). The study used the EpiData database for data entry and management, and the data entry was carried out using a double-entry verification. All statistical analyses were performed with SPSS software, version 26.0. For the non-normality of the data, a nonparametric signed-rank test was performed. The numerical variables were presented as the mean ± SD, and intragroup and intergroup comparisons were analyzed by the means of paired samples t-test and independent samples t-test, respectively. The categorical variables were described using the number of cases (%) and the Chi-square test was carried out. *p* values < 0.05 were considered statistically significant, and all tests were two-tailed.

## Results

### Characteristics of Patients

**FIGURE 1:**
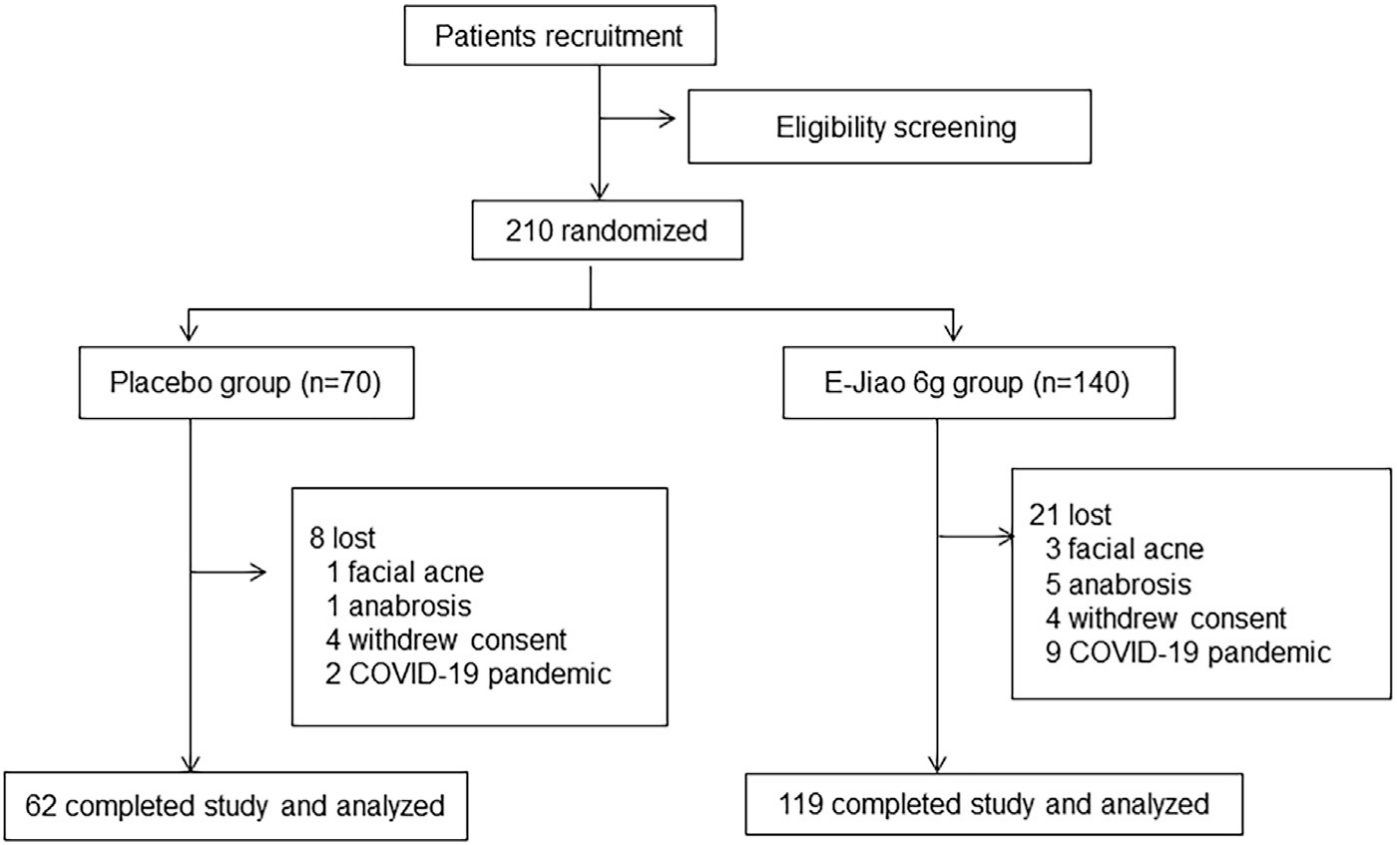
Trial flow chart primary recruitment identified 210 volunteers, the authors randomly allocated the patients to groups as follows: placebo (n = 70), Ejiao 3 g twice a day (BID) (n = 140).

According to the inclusion and exclusion criteria, 210 patients (Ejiao, n = 120; placebo, n = 70) were enrolled in the hospital. During the trial, 29 of the patients were lost. Of the 29 lost, 11 discontinued due to the COVID-19 pandemic and 18 for AEs such as facial acne and anabrosis. Finally, 181 patients completed the trial, 119 in the treatment group and 62 in the control group. The flow diagram of the trial is shown in Figure 1. Among the 210 recruited patients, 187 of them were women, while among completed patients, 163 out of the 181 patients were women. Therefore, we focused our analysis on the effect and safety of Ejiao in women with blood deficient symptoms. The general characteristics of the participants are shown in Table 1. The groups were well matched about baseline characteristics, including sex, age, body mass index, blood pressure, and heart rate (*p* > 0.05).

**TABLE 1:** Baseline characteristics of the study participants.

Characteristics	Ejiao	Placebo	*p*-values
Age (years)	37.99±13.50	39.63±13.21	0.436
Height (cm)	161.65±4.60	162.18±4.26	0.474
Weight (kg)	56.37±7.14	55.36±6.63	0.384
Body temperature (°C)	36.56±0.36	36.63±0.29	0.234
BMI (kg/m^2^)	21.61±2.42	21.27±2.53	0.569
Systolic blood pressure (mmHg)	110.84±9.33	108.47±10.16	0.674
Diastolic blood pressure (mmHg)	73.76±6.88	69.35±7.46	0.532
Heart rate	80.33±4.61	83.18±5.76	0.546

165 participants were randomly assigned to the placebo; Ejiao 3 g (BID). Data are expressed as mean ± standard deviation. *p*-values were calculated by independent samples t-test.

BID, twice a day; BMI, body mass index.

### Impact of Ejiao on TCM Syndromes

The severity of the symptoms were classified as “mild, moderate, and severe” according to the Guideline of Clinical Research of TCM New Drugs (Zheng, 2002). Our results indicated that after the treatment with Ejiao, the symptoms of dizziness were greatly alleviated. At enrollment, the majority of the patients were in the “mild” and “moderate group” and the remaining patients were in the “severe” (less than 5%) group. The baseline severity classification was similar between the two groups (Table 2). After treatment with either Ejiao or placebo, the frequency of “moderate” to “mild” patients gradually increased (Table 2); however, the change in the Ejiao group was much more obvious. After treatment for 56 days, the frequency of “moderate” in the Ejiao group decreased to 6.5%, which is significantly better than the control group (14.5%). The frequency of “severe” decreased from 4.6% to 0 in the Ejiao group, while no change was observed in the placebo group. These results indicate that Ejiao treatment resulted in superior improvements of dizziness classification compared with the placebo (*p* = 0.0043).

**TABLE 2:** The symptom of dizziness after administration of Ejiao for 8 weeks.

Time Point	Group	Mild	Moderate	Severe	*P*
0 days	Ejiao	58 (53.7%)	45 (41.7%)	5 (4.6%)	0.647
	Placebo	31 (56.4%)	23 (41.8%)	1 (1.8%)	
28 days	Ejiao	91 (84.3%)	17 (15.7%)	0 (0%)	0.918
	Placebo	46 (83.6%)	9 (16.4%)	0 (0%)	
56 days	Ejiao	101 (93.5%)	7 (6.5%)	0 (0%)	0.043
	Placebo	46 (83.6%)	8 (14.5%)	1 (1.8%)	

Data are expressed as the number of cases (%). *p*-values were analyzed by Chi-square test.

**FIGURE 2:**
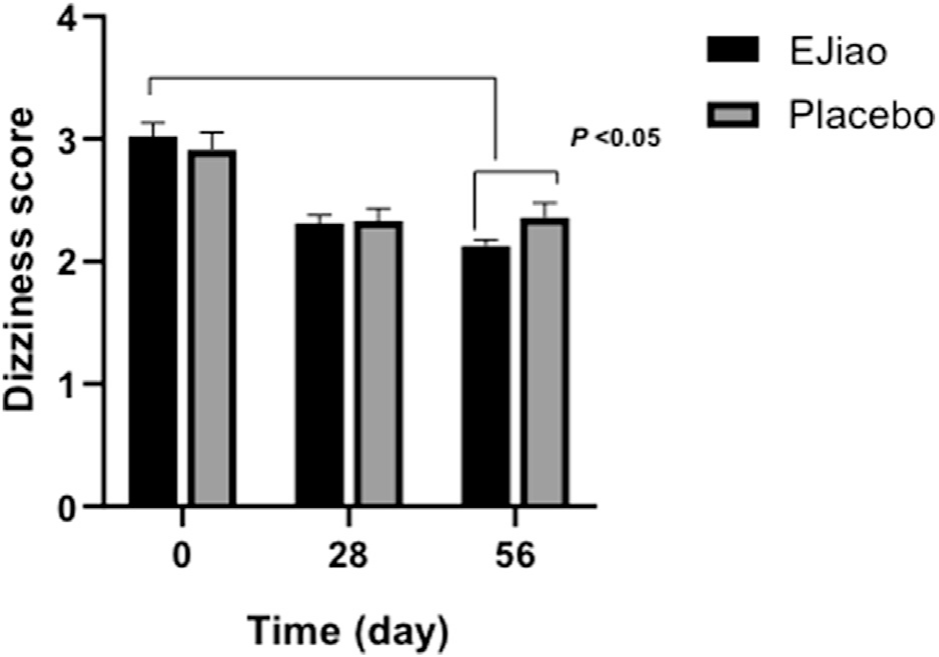
The dizziness syndrome scores of the Placebo and Ejiao groups were evaluated after treatment for 28 and 56 days. After 56 days of administration of Ejiao (3 g, BID), there was a significant improvement in the symptoms of dizziness in the Ejiao group compared to the Placebo group and pretreatment state. Data are expressed as mean ± standard deviation. Intragroup and intergroup comparisons are calculated by means of paired samples t-test and independent samples t-test, respectively.

The scores of TCM syndrome were also determined in our study. No differences were observed in the TCM syndrome scores between the two groups before treatment (3.02 ± 0.11 and 2.91 ± 0.14, respectively, *p* > 0.05). After treatment for 28 or 56 days, the scores decreased in both the Ejiao and placebo groups (Figure 2). All syndrome scores decreased significantly (*p* < 0.01) as compared with the baseline and more obviously after 56 days. After 4 weeks treatment with Ejiao, the scores decreased from 3.02 ± 0.11 to 2.31 ± 0.07, which further decreased to 2.13 ± 0.048 at the end of the trial. The scores in the Ejiao group showed statistical significance as compared to the control group. Other major syndromes of BDS, including pale complexion, palpitations, pale tongue, and thready pulse, were also evaluated in our study. However, Ejiao showed no significant improvement on these symptoms (Supplementary Figure 1). No significant difference was found in the secondary symptoms between the Ejiao and placebo groups after treatment (data not shown). The most possible reasons maybe that, first, in our study, we used only Ejiao instead of combinations, while in previous studies, Ejiao was usually combined with other TCM or Western drugs; second, the sample size is relatively small, and the treatment time is limited in our study, and more significant effects of Ejiao may be shown in studies with a bigger sample size and longer treatment time.

### Effects of Ejiao on Peripheral Blood Cells

**FIGURE 3:**
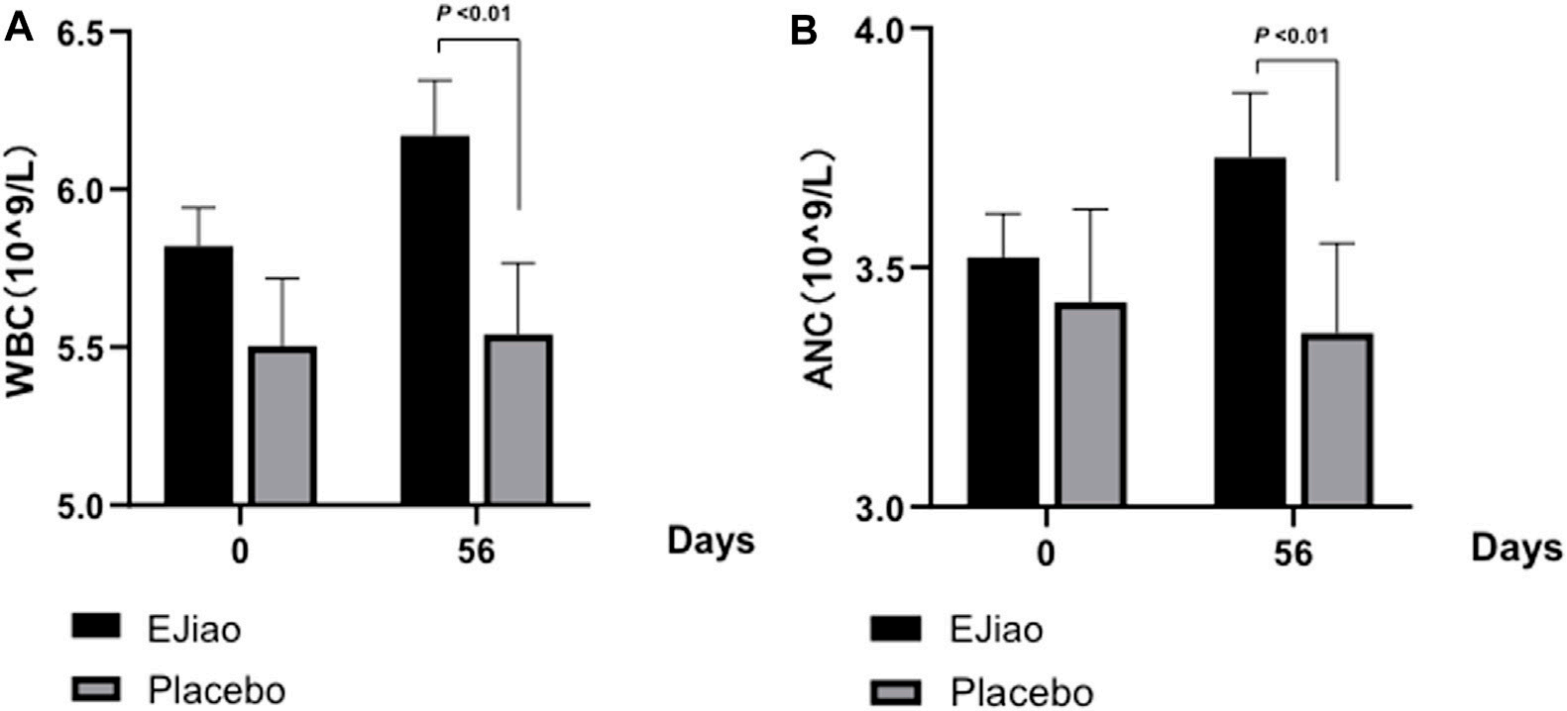
Changes in WBCs and ANC between the Ejiao and placebo groups in 56 days. After 56 days of administration of Ejiao (3 g, BID), the WBCs **(A)** and ANC **(B)** were significantly increased in the Ejiao group compared to the pretreatment stage and placebo group, while there were no significant changes in the placebo group after the treatment of Ejiao. The data are expressed as mean ± standard deviation. *p*-value is analyzed by independent samples t-test.

The quantities of different blood cells from the peripheral blood were investigated in our study. The results indicated that the decrease of RBC and hematocrit (HCT) were alleviated, while the levels of white blood cells (WBCs) and absolute neutrophil count (ANC) were increased (Table 3). During the clinical trial, although the red blood cell numbers decreased in both groups, the decrease was greater in the control group (Table 3). The difference of the changes between the two groups was also analyzed. Our results indicated that the RBCs decreased significantly in the placebo group (–0.13 ± 0.029) as compared with the Ejiao group (–0.043 ± 0.02). For the HCT, the number decreased greatly (−1.35 ± 0.27) in the control group, which was significantly alleviated in the Ejiao group (−0.57 ± 0.21, *p* = 0.036). The quantities of the WBCs and ANC showed no obvious change in the control group, while the treatment of Ejiao increased the WBCs and ANC, but still within the normal values during the observed time period (Figure 3).

**TABLE 3:** The changes of hematocrit and red blood cell numbers from the baseline over 56 days of treatment with Ejiao.

Item	Time point	Placebo	Ejiao		
		Outcome	Change from baseline	Outcome	Change from baseline
Hematocrit	Baseline	39.86±0.44	−1.35±0.27	39.11±0.28	−0.57 ± 0.21 (*p* = 0.036)
	56 days	38.41±0.41		38.42±0.32	
Red blood cell number	Baseline	4.41±0.042	−0.13±0.029	4.36±0.03	−0.043 ± 0.02 (*p* = 0.048)
	56 days	4.29±0.035		4.31±0.032	

Data are presented as mean ± SD. *p* values were calculated by independent samples t-test.

### Ejiao Improved the Quality of Life in BDS Patients

To evaluate the health status of participants, the SF-36, a self-assessment health status questionnaire, was constructed, which contains 36 items (questions) about sociodemographic data, health, and personal behavior, grouped into eight multi-item domains (Chen et al., 2020). The mean (±SD) differences between the groups in the outcome visits at the baseline, 4 weeks, and 8 weeks are shown in Table 4. The mean baseline differences showed no statistically significant difference between the two groups (*p* > 0.05). After treatment, there were statistically significant differences in role physical, vitality, social function, role emotional, and the health transition of SF-36 between the two groups at 4 weeks (*p* < 0.05).

**TABLE 4:** The changes of SF-36 subscales during the 24 weeks.

Item	Groups	Baseline	28 days	56 days	Change 28 days	Change 56 days
Role physical	Placebo (n = 55)	70.454.68	97.272.01	86.363.6	26.824.71	15.914.55
	Ejiao (n = 108)	63.893.62	97.450.80	82.872.87	33.563.79^*^	18.984.09
Vitality	Placebo (n = 55)	66.362.11	73.181.33	73.091.77	6.822.42	6.732.08
	Ejiao (n = 108)	60.321.66	72.081.02	71.301.39	11.761.94^**^	10.971.88^#^
Social function	Placebo (n = 55)	105.232.58	97.051.65	111.142.01	-8.193.16	5.912.48
	Ejiao (n = 108)	98.501.91	97.341.19	106.831.78	-1.162.27^**^	8.332.21
Role emotional	Placebo (n = 55)	58.795.39	94.181.03	86.064.12	35.395.26	27.275.67
	Ejiao (n = 108)	50.614.00	92.901.58	78.703.29	42.284.26^*^	28.094.66
Health transition	Placebo (n = 55)	36.572.4	41.672.80	52.782.76	5.093.90	16.203.50
	Ejiao (n = 108)	35.421.97	47.692.01	53.702.22	12.272.93^**^	18.292.60

Data are presented as mean ± SD. *p* values were calculated by independent samples t-test.

For the difference between the changes of Ejiao versus placebo during 28 days, **p < 0.01 compared with the control, *p < 0.05 compared with the control.

For the difference between the changes of Ejiao versus placebo during 56 days, #*p* < 0.05 compared with the control.

### Changes on Fireness

**FIGURE 4:**
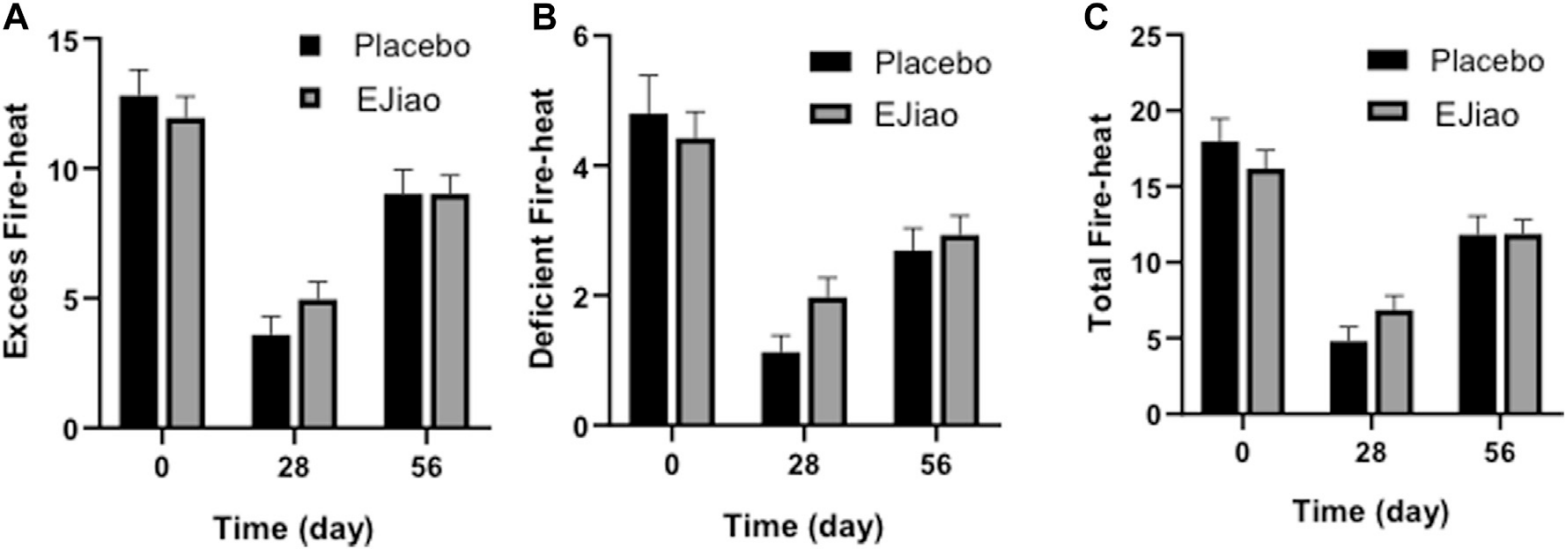
fire–heat syndrome scores of the placebo and Ejiao groups were evaluated before and 4 and 8 weeks after treatment. Syndrome score for total symptom item of excess fire–heat **(A)**, deficient fire–heat **(B)**, and total fire–heat **(C)** are expressed as mean ± standard deviation.

The fire–heat symptoms scale, including the excess fire–heat score (EFS) and deficient fire–heat score (DFS), was used in our study. The baseline in the placebo and Ejiao groups were 17.96 ± 1.50 and 16.19 ± 1.19 for the total fire–heat score; 12.80 ± 0.98 and 11.94 ± 0.82 for the EFS; and 4.80 ± 0.58 and 4.42 ± 0.40 for the DFS, respectively. As shown in Figure 4, the results showed that all the scores were significantly decreased at day 28, which then increased at the end of the study, which might contribute to the reason that the baseline was determined mainly during June to September in 2019, and the time point day 28 was mainly detected during October to February of the next year, while the time point day 56 was mainly detected during March to June. According to the TCM theory, fireness is closely related to the weather, and it is easier for people to get fireness syndromes in summer than in winter. As shown in Figure 4, the trend of the change were the same for both the Ejiao and control groups, and there was no significant difference between the two groups.

### Safety Evaluation

The types and occurrences of AEs during both the Ejiao and placebo treatments were evaluated. 11 AEs occurred during Ejiao treatment, while 4 AEs occurred during the placebo treatment (Table 5). However, this difference was not statistically significant. The most frequent AEs occurring during Ejiao treatment were anabrosis (n = 5) and facial acne (n = 3). No moderate or severe AEs were reported during the whole clinical trial. Compared with the placebo, there was no significant difference on the index of the kidney and liver functions (Cr, ALT, and AST levels) after treatment with Ejiao for 56 days between the two groups (data not included). The results of electrocardiograms were also within the normal range. These results indicate that Ejiao treatment for 56 days has no obvious adverse reactions on patients with BDS.

**TABLE 5:** Summary of AEs.

Item	Ejiao	Placebo	*p* value
Diarrhea	1 (0.9%)	1 (1.8%)	0.623
Sudden pneumothorax hospitalization	1 (0.9%)	0 (0.0%)	0.474
Poor appetite	2 (1.9%)	0 (0.0%)	0.309
Sudden herpes	0 (0.0%)	1 (1.8%)	0.159
Surgery for uterine fibroids	0 (0.0%)	1 (0.9%)	0.159
Facial acne	3 (2.78%)	1 (1.8%)	0.707
Anabrosis	5 (4.6%)	1 (1.8%)	0.364

Ejiao was administered (3 g twice a day [BID]) for 8 weeks. During the study period, all the AEs were documented. Data are expressed as the number of cases (%). *p* values were calculated by Chi-square test.

## Discussion

BDS is a traditional Chinese clinical term, which is related to anemia in modern medicine. BDS is more prevalent in women as compared to men. There may be several reasons for this: first, women suffer from chronic blood loss due to monthly menstrual cycles. This blood loss, if not corrected by an appropriate diet, always leads to chronic BDS; second, women’s hormones change at different stages of life, such as adolescence, pregnancy, lactation, and menopause. In all these stages, the demand for iron and calcium increases. If this increased demand is not met, they tend to suffer from BDS. Ejiao is thought to be the first choice for the treatment of BDS in women. In our clinical trial, although patients were recruited regardless of their sex, we found that among the 220 patients, 187 of them were females, which also indicated that Ejiao is more widely used in women. Therefore, the clinical study on safety and efficacy of Ejiao in females is important.

The hematopoietic effect of Ejiao has been well demonstrated from long-term clinical experience (Wang et al., 2014; Li et al., 2019). Studies have also proved that Ejiao promoted the recovery of hematopoietic function in 5-fluorouracil–induced blood deficiency in mice (Wu H. et al., 2007; Zhang Y. et al., 2019). Others have reported that the fractions from the enzyme-digested Ejiao might be the main active components, which are composed of different amino acids. In addition, the effect of Ejiao has also been compared with other traditional Chinese medicines. The leukocytopoiesis-promoting action of effective components from *Spatholobus suberectus Dunn* (Leguminosae; *Caulis spatholobi*) and Ejiao in rats with leukopenia induced by cyclophosphamide was compared, the results showed that the effective components from *C. spatholobi* could significantly promote leukocytopoiesis in rats, and the effect was equivalent with that of Ejiao (Ying et al., 2011). The effect of formulae containing Ejiao was also compared, the study showed that the formula containing Ejiao and *Ganoderma sinense* Zhao, Xu et Zhang (Polyporaceae; Ganoderma) had a better effect on the complex blood-deficient model, while the formulae containing *Angelica sinensis* (Oliv.) Diels (Umbelliferae; Angelicae Sinensis Radix), and *Astragalus membranaceus* (Fisch.) Bge. var. *mongholicus* (Bge.) Hsiao (Leguminosae; Astragali Radix) showed better effects on the blood losing model (Chen et al., 2015).

Although these researches are important to prove the functions of Ejiao and explore the underlying mechanisms, the evidence from the clinical trials is more direct and convincing. In clinical practice, Ejiao is usually used with a combination of other traditional Chinese medicine according to different constitutions and symptoms of the patients. Therefore, the randomized, double-blind, placebo-controlled trials were focused to explore the safety, effectiveness, and cost-effectiveness of the formulae which contained Ejiao (Zhang Y. et al., 2019). Our study is the first clinical study to provide systematic evidence for the blood nourishing effect of Ejiao in female patients with BDS. And here, we showed that the major symptoms of BDS were greatly improved after the treatment with Ejiao. The reduction of RBCs was controlled, while the numbers of WBCs were increased, which are consistent with previous *in vitro* studies. Our research provided scientific foundation for the application of Ejiao in BDS patients.

Fireness is a special term in TCM without objective diagnostic parameters (Lin et al., 2012; Xu and Dou, 2016). It is commonly thought that some of the traditional Chinese medicines can cause fireness, such as ginseng, Ejiao, and so on. Our previous study has already investigated whether the use of red ginseng, another famous traditional medicine used for thousands of years, would cause fireness. Our results showed that the proper use of ginseng in patients with deficiency syndrome is safe and will not cause fireness. Similar to ginseng, Ejiao is also a tonic medicine. Many people believe that the use of it can cause fireness, especially in summer. Although some studies reported that Ejiao could cause fireness, no systematic clinical trial has been conducted to study the direct relationship between Ejiao and fireness. Our study here is the first randomized, double-blind, placebo-controlled clinical trial to study the safety of Ejiao in female patients with BDS. Our results demonstrated that the fireness symptom scores showed no significant changes in the Ejiao group. Facial acne and anabrosis were thought to be symptoms related to fireness, although these cases were reported more in the Ejiao groups than in the placebo control groups, and statistical analyses showed that there were no significant differences between the two groups. Our data indicate that there is no safety concern with the consumption of Ejiao in female BDS patients.

There are some limitations in our study. First, our study only recruited participants with BDS. However, many people take Ejiao without considering about their constitution. Therefore, the results here cannot be generalized to all Chinese people. However, according to the TCM theory (Yi et al., 2017; Li et al., 2018), Ejiao is only suitable for patients with BDS. Therefore, it is more reasonable to choose patients with BDS as the research object. Second, 11 cases were lost and fell off due to the COVID-19 pandemic. However, we managed to control the cases lost within 20%. We took the questionnaire on the Internet or by means of telephone calls to reduce the risk of loss to follow-up. We delivered the research materials to participants by mail to maintain the trial. Third, we only studied the efficacy of Ejiao in our study, but the mechanisms of it have not been studied. However, our main objective was to provide direct evidence for the use of Ejiao in BDS patients, and our results have indicated that the major symptom of BDS was improved. Further studies will be conducted to elucidate the mechanism of the effect in the future.

In summary, the safety and efficacy of Ejiao in participants with BDS were first evaluated. Our results showed that Ejiao greatly alleviated the syndrome of dizziness, which is one of the major syndromes of BDS. The impact of Ejiao treatment on the peripheral blood indicated that Ejiao alleviated the decrease of RBC and HCT, while it increased the levels of WBCs and ANC. Safety evaluation indicated that Ejiao treatment was not associated with an increase in the fire–heat symptom score for people with BDS.

## Data Availability

The original contributions presented in the study are included in the article/Supplementary Material; further inquiries can be directed to the corresponding authors.
