# Supplementary figures and images for: Efficacy and Safety of Ejiao (Asini Corii Colla) in Women With Blood Deficient Symptoms: A Randomized, Double-Blind, and Placebo-Controlled Clinical Trial
Source: Front Pharmacol. 2021 Oct 11;12:718154. doi: 10.3389/fphar.2021.718154 (PMC8542698; doi:10.3389/fphar.2021.718154)

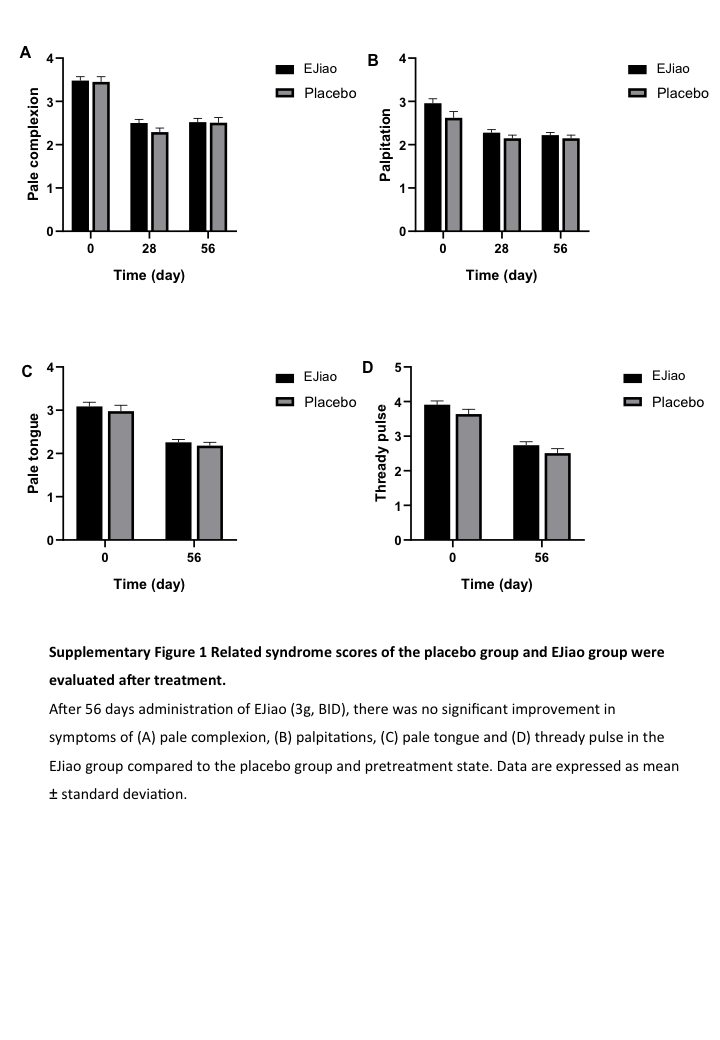

Supplement: Supplementary file 1 [file Image1.TIFF]
